# From multivariate methods to an AI ecosystem

**DOI:** 10.1038/s41380-021-01116-y

**Published:** 2021-05-12

**Authors:** Nils R. Winter, Micah Cearns, Scott R. Clark, Ramona Leenings, Udo Dannlowski, Bernhard T. Baune, Tim Hahn

**Affiliations:** 1 Department of Psychiatry, University of Muenster, Münster, Germany; 2 Discipline of Psychiatry, School of Medicine, University of Adelaide, Adelaide, SA Australia; 3 Department of Psychiatry, Melbourne Medical School, The University of Melbourne, Parkville, VIC Australia; 4 The Florey Institute of Mental Health, The University of Melbourne, Melbourne, VIC Australia

**Keywords:** Predictive markers, Psychiatric disorders

A decade ago, at a major international conference, we vividly remember a symposium on psychiatric Artificial Intelligence (AI) drawing a crowd of seven people—including the four speakers. Today one might get the impression that every other funding proposal is required to include at least some degree of AI-based analyses. At a time when 17 of the 29 hot topics listed by the most recent *Gartner Hype Cycle* for emerging technologies [1]—an indicator of perceived innovation—are either AI technologies (e.g., Generative Adversarial Networks) or include AI as a core component (e.g., autonomous driving), there is no shortage of promises. In both psychiatry and medicine in general, expectations to move beyond classical group-level statistics and enter the promising future of personalized medicine are high. Although AI has not yet fully hit mainstream psychiatric research, the availability and advancement of technology and methods have indeed led to a growing adoption of AI methods and agreement on best practice [2–4]. Despite this progress and some promising first applications (e.g., in suicide prediction [5]), translation to clinical practice has been hampered by a large degree of estimate variability and diagnostic heterogeneity.

In the following, we will argue that the current drawbacks in psychiatry arise not primarily from a lack of methodological advancement and genuine clinical potential for AI in psychiatry, but from fundamental issues pertaining to sample size, model construction, evaluation practice, and the conceptualization of mental disorders. To overcome these challenges, we outline concrete steps towards an AI ecosystem that promotes a coordinated collaboration within the field to pave the way for better translation of AI solutions into clinical practice (see Fig. 1). Built upon four supporting pillars; collection, construction, evaluation, and translation, this ecosystem provides a framework for data collection, harmonization and sharing, guidelines for model construction, evaluation and distribution, methods for parsing heterogeneity as well as ethical and transparency standards.

**Fig. 1:**
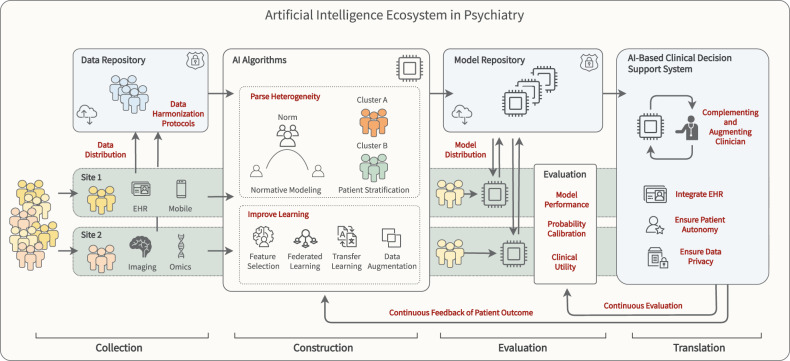
Artificial intelligence ecosystem in psychiatry. Outline of an AI ecosystem with four supporting pillars: collection, construction, evaluation, and translation. Exemplarily, two clinical sites are shown in light green in the bottom row. Top row illustrates mainly off-site, cloud-based infrastructure in light blue.

## Explaining the translational roadblock

In the social and medical sciences, in light of the recent replication crisis, a failure to replicate results has rendered many discoveries from group-based statistical analyses void [6]. While machine learning’s goal of optimizing model performance for previously unseen data integrates replication directly into model development, which in theory, should render it more robust to replication failure, analyses drawing on larger samples has revealed substantial overestimation of model performance for many landmark studies. For example, at a recent international machine learning competition, participants sought to classify major depressive disorder (MDD) patients from healthy controls using structural Magnetic Resonance Imaging data (*N* participants = 2240) [7]. In contrast to numerous smaller studies showing accuracies of more than 80–90%, the winning entry ceiled at only 65% accuracy. This effect was demonstrated again on a large structural imaging sample from the UK biobank (*N* > 3400), finding accuracies of only 50–60% across classification targets [8].

These findings lead to two important questions. Firstly, where in the analysis pipeline are we most susceptible to overestimating model performance in small sample size studies? Secondly, are these overestimations and subsequent replication failures distinct to structural imaging studies or should we expect to see this same phenomenon with other data modalities, also? A recent study empirically addressed both points [7]. First, the authors demonstrate that predictive structural imaging studies of MDD are prone to systematically overestimating model performance on smaller samples. Second, this effect arises not primarily from small training samples, but is due to too small test sets. Third, this statistical effect is generalizable and contingent on the cross-validation cycle itself rather than the phenotype or data modality used for model training. Thus, the results are likely to generalize across all areas of psychiatric research (from models using imaging, to proteomics, and to routine clinical data).

In addition to these statistical properties, the translational abilities of any AI model are dependent upon the clinical utility and construct validity of a diagnostic category, its related illness trajectories, and the degree of heterogeneity contained within each. This latter point partially explains why smaller more homogeneous test samples overestimate the true predictive ability of a model compared to accuracy estimates attained on larger more heterogeneous samples reflective of the true patient population [9]. Therefore, it is likely that the underlying causes of therapeutic response and illness trajectories vary qualitatively and quantitatively between different, more homogeneous subsamples of these categories. Outside of applied AI, this is already recognized as a major obstacle to translatable clinical research and has sparked many theoretical considerations such as the *Research Domain Criteria*, the *Roadmap for Mental Health Research in Europe*, or the *Hierarchical Taxonomy of Psychopathology*. These problems cannot be eliminated easily but require novel approaches to the classification and stratification of psychiatric disorders, including e.g., normative modeling approaches [10] (see *Construction* below).

## Establishing an AI ecosystem

### Collection

While often dismissed as too costly and impractical, feasibility of large-scale data acquisition heavily depends on data modality and existing infrastructure. For example, systematically making routine patient data and electronic health records available will enable dramatic increases in sample size and coverage of potential patient subpopulations, thereby outweighing the often lower quality of this data. In the same vein, building on high-throughput omics platforms or mobile sensing technology (e.g., from smartphones and watches) will allow us to cost-efficiently gather ecologically valid physiological and phenotype data in real-time. We have already seen the great efficacy of data sharing within large consortia in genetics (e.g., PGC) and neuroimaging (e.g., ENIGMA). Similar initiatives and protocols have not yet been established for EHR and eHealth data but will be integral to an AI ecosystem. In cases where data remains limited due to scarcity of the outcome or patient group (e.g., patient subgroups with certain comorbidities), harmonizing data acquisition protocols will be key to training machine learning models based on data from multiple sources—especially with regard to the reduction of epistemic uncertainty. Wherever acquisition protocols differ, we must ensure the development of methods for harmonizing the data itself. However, as data collection is increasingly organized through large initiatives such as the UK Biobank, we must not reduce our efforts in establishing more and secure infrastructure to upload and share data from small studies. This is important since smaller studies often investigate important mental disorders, comorbidities with low incidence, or causal mechanisms that we are less likely to capture in large-scale consortia. Studies by Chekroud et al. [11] or Miotto et al. [12] show the great potential large but clinically relevant samples can have but as of now, those are the exception rather than the rule, and similar efforts are needed across all areas of psychiatry.

### Construction

In addition to collecting more data, the issue of sample size can also be dealt with at the model construction stage. This is especially relevant when dealing with highly specific questions (e.g., a distinct type of psychotherapeutic intervention) or low-incidence disorders where we cannot collect large amounts of data. Methods for dealing with small training samples have been discussed in previous works [4]. To summarize, one might try to generate synthetic training data through data augmentation or leverage larger training samples from another task through transfer learning. Additionally, formally incorporating (causal) domain knowledge gained from basic research directly (e.g., selecting features that have shown to be relevant for a specific task across studies through meta-analytic approaches [13]) into machine learning models also holds great promise to dramatically decrease the number of training samples required [14]. In addition, federated or collaborative learning allows us to train an algorithm across multiple sites by passing a model trained on one site to the next for further training without the need to exchange sample data, thereby alleviating data protection issues whilst maximizing training sample size. This approach is especially appealing at a time when the importance of protecting sensitive data is increasingly recognized. Easy-to-use platforms like Coinstac [www.coinstac.org] or DataSHIELD [www.datashield.ac.uk] that allow federated learning have started to emerge and will integrate nicely into an AI ecosystem.

Also crucial for an AI ecosystem are ways in which we can parse population heterogeneity. From a methodological perspective, we can differentiate two approaches. First, traditional clustering methods can reveal more homogeneous patient subgroups. Depending on the clustering approach and data used, subjects in these groups might share specific symptoms, a common genotype, or a similar neurobiology that is clinically informative yet evades symptom-based diagnostic categories. At the symptom level, some studies already suggest that symptom clustering might enhance the prediction of antidepressant treatments [15]. While easily lending itself to a transdiagnostic view, jointly modeling biological and deep phenotype data with multi-view methods such as Canonical Correlation Analysis or Partial Least Squares Regression show promise for uncovering transdiagnostic modes of covariation between biological data and behavioral phenotypes. Embedding these low-dimensional representations within supervised prediction frameworks may assist in overcoming population heterogeneity and help decrease our dependency on large training and testing samples.

Second, normative modeling [10] quantifies the degree to which individuals deviate from the expected population distribution, thus, allowing symptoms in individual patients to be recognized as extreme values within a distribution. Unlike clustering approaches, normative modeling does not require a consistent pattern of deviation and can therefore accommodate for the heterogeneity across patients. While normative modeling provides a highly effective and principled approach to the quantification of deviation, a direct translation to clinically useful machine learning models must still be developed.

Finally, clinically useful predictive models need to provide uncertainty estimations for their predictions. We not only need to know how accurate a model is, but how confident it is in its prediction, and whether or not this confidence aligns with the true probability of an event occurring. For example, imagine a model that is highly accurate and deployed in a clinic. It predicts that a high-risk patient will not respond to a first line treatment, but instead, to a first-generation treatment with a high side-effect profile. In this situation, a clinician needs to make a nuanced decision that weighs up the associated risks against the potential benefits of the treatment. If a model turns out to be only 51% confident in its prediction, even though on average it is 80% accurate, the weighting that a clinician should give to the model should not be high. However, many models currently used in psychiatry (e.g., support vector machines, random forests etc) are poorly calibrated by virtue of their underlying mathematics, meaning that they may be highly confident in their decision when the true probability of an event occurring in the population is actually not much better than chance. This is a seldom discussed issue in computational psychiatry with far reaching implications for clinical decision making [16].

### Evaluation

As one of the fundamental problems of overestimating model performance has its roots primarily in the evaluation framework itself, a concise AI ecosystem needs to provide guidelines and an independent infrastructure to adequately evaluate model performance. While the Focus Group on artificial intelligence for health (FG-AI4H) has begun to outline a standardized assessment framework for the evaluation of AI models in partnership with the World Health Organization, simple online model-sharing platforms already enable the upload of machine learning models for evaluation by others (e.g., the PHOTON AI model repository; www.photon-ai.com/repo). Extending such initiatives into largely automatized AI assessment infrastructure would allow for highly efficient and transparent model evaluation at scale. To foster transparency, journals and funding agencies ought to ensure that all code is available on request or provided in an online repository upon submission. Finally, the increasing availability of easy-to-use, open-source machine learning frameworks (e.g., the mlr3 R package [mlr3.mlr-org.com], the PHOTON AI framework [www.photon-ai.com] or tidymodels [www.tidymodels.org/]) will continue to simplify robust and replicable machine learning analysis.

In addition to performance-based evaluation (e.g., model accuracy), we also need to assess the clinical utility of a model through practical questions. For example, can we easily use the model without data protection issues? Does the model provide uncertainty estimates and calibrated predictions? Is the model clinically useful, i.e., does it provide us with information we cannot otherwise obtain and/or outperform clinicians in the field? Does the model provide information that effectively supports and augments the clinician? For example, will a costly case/control biomarker model trained on *diagnostic* labels that are already provided by clinicians actually tell them anything that improves their clinical decision-making process? In situations where biomarkers are of *prognostic/predictive* value, above and beyond that of cheap and easily accessible routine clinical data, is the increase in prognostic certainty justified by the increased health economic costs and invasiveness to obtain these markers at the individual patient level? Overall, questions and measures of clinical utility should be asked and assessed in the same way that we examine a model’s predictive performance. Incorporating clinical utility-based evaluation into existing guidelines will be fundamental to progress from proof-of-concept studies to clinical translation.

### Translation

Moving predictive models from bench to bedside can be accomplished in a number of ways. Online risk calculators are simple and cost-effective ways of supporting patient care and have already been used for the detection of individuals developing psychosis in secondary mental health care [17]. More complex predictive models can be provided through the same infrastructure that is already used for model evaluation. Patients’ data could be uploaded to these online models which in turn provide individual predictions. Whenever data privacy is a concern, those models need to also be made available offline. Ultimately, predictive models should be incorporated into AI-based clinical decision support systems (CDSS) for integration into a clinician’s workflow. If designed properly, these AI-based CDSS could also facilitate continuous evaluation through feedback loops whilst also improving model performance over time. In addition, providing predictive algorithms with an *individual* patient’s long-term outcomes and symptom developments would signify a major step towards parsing diagnostic and population heterogeneity. Importantly, as the role of AI in medical care expands, we need to establish ethical standards that balance patient autonomy and foster clinician and patient trust.

## Conclusion

Against the background of fundamental issues regarding sample size, model construction, evaluation practice, and the conceptualization of mental disorders, the clinical success of AI in psychiatry will depend on the timely creation of an AI ecosystem that addresses these foundational issues hampering the validity and reliability of the current literature. For the field to emerge from the hype, through the trough of disillusionment and into a plateau of productivity, we propose four foundational pillars to bolster the field. First, building upon existing data collection initiatives and consortia, we need to address the issue of small training samples by incentivising researchers to harmonize protocols and data acquisition, not only making sure to collect bigger data, but more clinically relevant data for model training. Second, at the model construction phase we need to take advantage of methods such as transfer learning and data augmentation that mitigate some of the detrimental effects of small sample sizes. A simple, safe, and open-sourced federated learning infrastructure would instantly help overcome this problem as it would allow for the training of AI models without the need to share data. In addition, transdiagnostic clustering and normative modeling approaches may provide a framework to address the long-standing issue of diagnostic heterogeneity. Further, using algorithms that are well calibrated and thus able to provide estimates of uncertainty are of crucial importance if to ever be deployed clinically. Third, we must ensure reproducibility of machine learning analyses through the use of code sharing and model evaluation infrastructure. Extending existing model repositories would enable large-scale, independent model evaluation and standardized guidelines for rating the clinical utility of AI solutions. This will help ensure their alignment with the needs of patients and clinicians outside of the academic community. Fourth, integrating models into AI-based CDSS will help transform the way we treat and diagnose patients. Ensuring data privacy and patient autonomy at this level is of utmost importance to generate trust toward this new technology. Importantly, in all areas, leading consortia and machine learning groups show that many of the solutions already exist and are actively developed. Building an AI ecosystem based on these advances has grown from a daunting challenge into a unique opportunity for psychiatry in the decade to come.

## References

[1] Panetta K. The Gartner Hype Cycle highlights the 29 emerging technologies CIOs should experiment with over the next year. 2019. https://www.gartner.com/smarterwithgartner/5-trends-appear-on-the-gartner-hype-cycle-for-emerging-technologies-2019.

[2] Hahn T, Nierenberg AA, Whitfield-Gabrieli S (2017). Predictive analytics in mental health: applications, guidelines, challenges and perspectives. Mol Psychiatry.

[3] Poldrack RA, Huckins G, Varoquaux G (2020). Establishment of best practices for evidence for prediction: a review. JAMA Psychiatry.

[4] Cearns M, Hahn T, Baune BT (2019). Recommendations and future directions for supervised machine learning in psychiatry. Transl Psychiatry.

[5] Kessler RC, Bossarte RM, Luedtke A, Zaslavsky AM, Zubizarreta JR (2020). Suicide prediction models: a critical review of recent research with recommendations for the way forward. Mol Psychiatry.

[6] Stupple A, Singerman D, Celi LA (2019). The reproducibility crisis in the age of digital medicine. Npj Digit Med.

[7] Flint C, Cearns M, Opel N, Mehler D, Emden D, Leenings R, et al. Systematic overestimation of machine learning accuracies in structural neuroimaging studies of depression. ArXiv Prepr. 2019. https://arxiv.org/abs/1912.06686.

[8] Stolicyn A, Harris MA, Shen X, Barbu MC, Adams MJ, Hawkins EL, et al. Automated classification of depression from structural brain measures across two independent community-based cohorts. Hum Brain Mapp. 2020;41;3922–37. 10.1002/hbm.25095 PMC7469862 32558996

[9] Schnack HG, Kahn RS (2016). Detecting neuroimaging biomarkers for psychiatric disorders: sample size matters. Front Psychiatry.

[10] Marquand AF, Kia SM, Zabihi M, Wolfers T, Buitelaar JK, Beckmann CF (2019). Conceptualizing mental disorders as deviations from normative functioning. Mol Psychiatry.

[11] Chekroud AM, Zotti RJ, Shehzad Z, Gueorguieva R, Johnson MK, Trivedi MH (2016). Cross-trial prediction of treatment outcome in depression: a machine learning approach. Lancet Psychiatry.

[12] Miotto R, Li L, Kidd BA, Dudley JT (2016). Deep patient: an unsupervised representation to predict the future of patients from the electronic health records. Sci Rep.

[13] Karrer TM, Bassett DS, Derntl B, Gruber O, Aleman A, Jardri R (2019). Brain-based ranking of cognitive domains to predict schizophrenia. Hum Brain Mapp.

[14] Schölkopf B. Causality for machine learning. ArXiv. 2019. https://arxiv.org/abs/1911.10500.

[15] Chekroud AM, Gueorguieva R, Krumholz HM, Trivedi MH, Krystal JH, McCarthy G (2017). Reevaluating the efficacy and predictability of antidepressant treatments: a symptom clustering approach. JAMA Psychiatry.

[16] Cearns M, Hahn T, Clark S, Baune BT (2020). Machine learning probability calibration for high-risk clinical decision-making. Aust N Z J Psychiatry.

[17] Fusar-Poli P, Rutigliano G, Stahl D, Davies C, Bonoldi I, Reilly T (2017). Development and validation of a clinically based risk calculator for the transdiagnostic prediction of psychosis. JAMA Psychiatry.

